# DNA methylation is involved in sexual differentiation and sex chromosome evolution in the dioecious plant garden asparagus

**DOI:** 10.1038/s41438-021-00633-9

**Published:** 2021-09-01

**Authors:** Shu-Fen Li, Can-Can Lv, Li-Na Lan, Kai-Lu Jiang, Yu-Lan Zhang, Ning Li, Chuan-Liang Deng, Wu-Jun Gao

**Affiliations:** College of Life Sciences, Henan Normal University, Xinxiang, 453007 China

**Keywords:** Plant reproduction, DNA methylation

## Abstract

DNA methylation is a crucial regulatory mechanism in many biological processes. However, limited studies have dissected the contribution of DNA methylation to sexual differentiation in dioecious plants. In this study, we investigated the variances in methylation and transcriptional patterns of male and female flowers of garden asparagus. Compared with male flowers, female flowers at the same stages showed higher levels of DNA methylation. Both male and female flowers gained DNA methylation globally from the premeiotic to meiotic stages. Detailed analysis revealed that the increased DNA methylation was largely due to increased CHH methylation. Correlation analysis of differentially expressed genes and differentially methylated regions suggested that DNA methylation might not have contributed to the expression variation of the sex-determining genes *SOFF* and *TDF1* but probably played important roles in sexual differentiation and flower development of garden asparagus. The upregulated genes *AoMS1*, *AoLAP3*, *AoAMS*, and *AoLAP5* with varied methylated CHH regions might have been involved in sexual differentiation and flower development of garden asparagus. Plant hormone signaling genes and transcription factor genes also participated in sexual differentiation and flower development with potential epigenetic regulation. In addition, the CG and CHG methylation levels in the Y chromosome were notably higher than those in the X chromosome, implying that DNA methylation might have been involved in Y chromosome evolution. These data provide insights into the epigenetic modification of sexual differentiation and flower development and improve our understanding of sex chromosome evolution in garden asparagus.

## Introduction

As an essential epigenetic regulatory mechanism in eukaryotic species, DNA methylation contributes greatly to transposon silencing, heterochromatin organization, genome integrity maintenance, and gene expression regulation^1–4^. In higher plant genomes, DNA methylation usually exists in CG, CHG, and CHH (where H represents A, T, or C) sequence contexts^5^. The genomes of different plants show distinct DNA methylation profiles^6^. Moreover, the DNA methylation ratio and level change dynamically in different plant tissues at diverse developmental stages^7,8^. Accumulating reports have suggested that DNA methylation is a key modulator of numerous aspects of plant development, such as fruit ripening^7,9^, seed germination and development^8^, and stress responses^10^. However, the possible role of DNA methylation in sexual differentiation and unisexual flower development in dioecious plants is largely unclear.

The dioecious system rarely occurs in flowering plants, in contrast to the phenomenon in animals. Only approximately 6% of angiosperm species are dioecious; that is, each individual harbors only male or female flowers^11^. Dioecious plants are believed to have evolved from hermaphroditic species numerous times^12,13^. Most dioecious plants carry sex chromosomes, which are well documented to be derived from autosomes. Sex chromosome evolution process involves several developmental events, such as recombination suppression, repeat sequence accumulation, and Y chromosome heterochromatization and degeneration^14^. It has been revealed that DNA methylation may play a role in sex chromosome degeneration^15^. The determination and differentiation of opposite sexes are usually genetically governed by sex-determining genes located on sex chromosomes^14^. The sexes of plants are usually reflected in the flowers, which are formed during the reproductive stage. Sex determination/differentiation and flower development processes are regulated by dynamic networks among a variety of genes, transcription factors (TFs), and other modulators, including microRNAs and epigenetic modifications^16–18^. Recent studies have identified several sex-linked candidates sex-determining genes in a few dioecious plants^19–22^. The potential functions of these genes vary dramatically due to the independent origins of dioecious plants. Moreover, many sex-biased genes involved in sexual differentiation and flower development have been detected by using comparative transcriptome sequencing and other traditional methods^16,23–25^.

Garden asparagus (*Asparagus officinalis*) is a dioecious plant species, and its sex is controlled by X and Y sex chromosomes. It has a 1 C genome size of 1.3 Gb and 2*n* = 2*x* = 20 chromosomes^26,27^. Genome sequencing of garden asparagus has enabled assembly of 986 Mb of the genome, including a small male-specific region of the Y chromosome (MSY, 847 kb)^20^. Two genes located in MSY have been detected as candidates for sex determination: one is a potential female suppression gene (*SOFF*), and the other (*TDF1*) may function as a male activator^20,28^. In addition, a number of sex-biased genes have been detected and are potentially involved in sexual differentiation and flower development processes^24,25^.

The present study aimed to determine whether DNA methylation influences the transcriptional levels of sex-biased genes and whether it is involved in sex determination, sexual differentiation, and flower development processes of garden asparagus. A detailed analysis of the garden asparagus methylome and transcriptome was carried out using male and female flowers at different developmental stages. The results can provide valuable information for understanding the DNA methylation landscape and epigenetic regulation of sexual differentiation and flower development in garden asparagus.

## Materials and methods

### Plant material and sample preparation

Plants of the garden asparagus variety ‘UC309’ were grown in the experimental plot of Henan Normal University. The sex of the garden asparagus was determined by the floral phenotype. The individuals used in this study were progenies of one male and one female plant. The flower buds of males (six plants) and females (six plants) at the premeiotic (0.5–0.7 mm in length) and meiotic (1.0–1.6 mm in length) stages were separately pooled and immediately frozen in liquid nitrogen. The premeiotic and meiotic stages were determined by referring to published articles^25,29^ and cytogenetic analysis. Genomic DNA from flower buds was isolated using the traditional cetyltrimethylammonium bromide method. Total RNA from asparagus flower buds (three biological replicates per sample) was isolated using TRIzol reagent (Life Technologies, CA, USA).

### Whole-genome bisulfite sequencing (BS-seq)

After DNA concentration and integrity were detected, the DNA libraries for BS-seq were prepared from 500 ng of genomic DNA, which was sonicated into 100‒300 bp fragments. The fragmented DNA was purified and then subjected to end-blunting, A-extension, and adaptor ligation. For bisulfite treatment, an EZ DNA Methylation-Gold^TM^ Kit (Zymo, CA, USA) was used. The converted DNA fragments were subsequently amplified and then subjected to sequencing via the Illumina HiSeq™ 2500 platform.

### DNA methylation data analysis

The clean reads were mapped to the garden asparagus genome with the BSMAP program (version 2.90)^30^ using the default settings. Thereafter, a custom Perl script was used to identify methylated cytosines, and the methylated cytosines were tested as described in Lister et al^31^.

Differentially methylated regions (DMRs) between different comparison groups were identified using Pearson’s chi-square test (χ^2^) in methylKit (version 1.7.10)^32^. We first partitioned the genome into 200 bp regions with overlaps of 100 bp. Then, DMRs (*FDR* ≤ 0.05) were identified with methylation differences of 0.25, 0.25, and 0.15 for CG, CHG, and CHH, respectively.

### RNA-seq and data analysis

RNA-seq was conducted as previously described^25^. After sequencing and filtering, the clean reads were rRNA-depleted using Bowtie2^33^ and then mapped to the garden asparagus genome with TopHat2 (version 2.0.3.12)^34^ with the parameter settings *-g 1 -no-coverage-search -r 50 -mate-std-dev 80*. Transcript abundance quantification was performed by using RSEM^35^. Differentially expressed genes (DEGs) of different comparison groups were determined with the criteria of a fold-change ≥ 2 and a *q* value < 0.05.

### DNA methylation and gene expression correlation analysis

The annotated genes were first classified into four groups on the basis of gene expression levels: nonexpressed (none) (FPKM value ≤ 1), weakly expressed (low) (1 < FPKM ≤ 10), moderately expressed (middle) (10 < FPKM ≤ 100), and highly expressed (high) (FPKM > 100) genes. The methylation levels in the gene body and adjacent regions of these groups were determined. DMR-associated DEGs were analyzed by identifying DEGs harboring DMRs in the three contexts within the promoter, gene body, and downstream 2 kb regions.

## Results

### DNA methylation landscape of garden asparagus flower buds

**Fig. 1:**
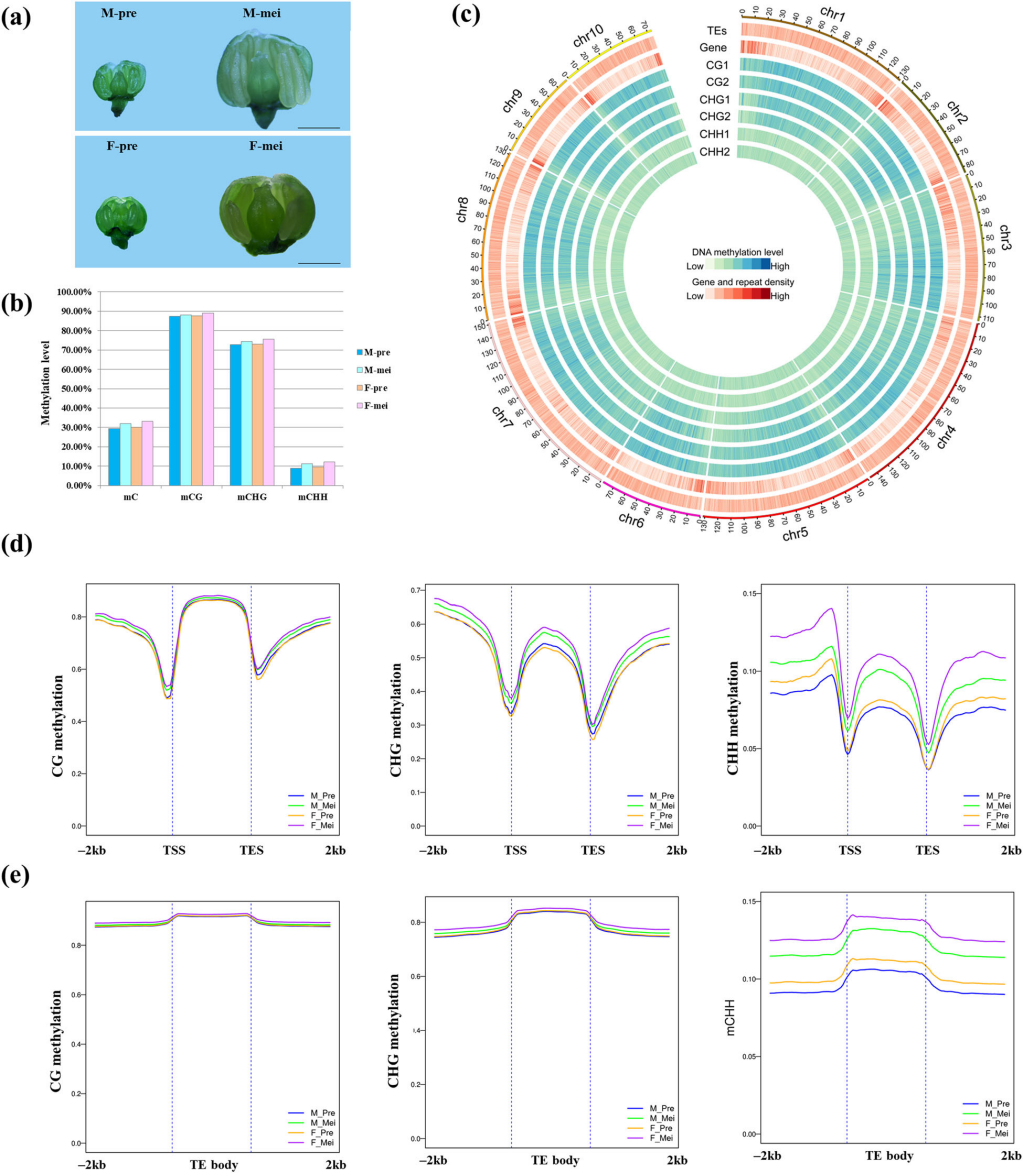
DNA methylation characterization of garden asparagus. **a** Morphological comparison of male and female flower buds of garden asparagus at different developmental stages. **b** Mean DNA methylation levels of C, CG, CHG, and CHH in different samples. **c** Overview of DNA methylation in 10 chromosomes. **d** DNA methylation profiles in gene bodies and adjacent regions in the three sequence contexts. **e** DNA methylation characteristics of TEs and upstream/downstream regions in each context

We conducted a whole-genome BS-seq of premeiotic and meiotic flower buds of males and females (Fig. 1a). A total of 1.05 billion clean single-end 150 bp reads were generated, and BS-seq had an average sequencing depth of 29.06 × per sample. On average, approximately 73.6% of cytosines of the garden asparagus reference genome sequence were covered by one or more reads (Supplemental Fig. 1).

A total of ~314 and 312 million mCs in male premeiotic and meiotic flower buds and ~317 and 310 million mCs in female premeiotic and meiotic flower buds were identified. After at least four-read coverage filtering, ~145.8 million methylated cytosine sites in each sample were identified on average, accounting for 46.54% of the cytosines with the reference genome. Generally, the methylation patterns among the four samples were similar (Fig. 1b). We used male premeiotic stage flower buds as representative samples for methylation pattern analysis. As shown in Fig. 1c, the CG and CGH methylation sites were highly abundant in gene-poor heterochromatin regions with high transposable element (TE) frequencies, whereas CHH methylation displayed a weak and uniform distribution, which is consistent with observations in other plant species^36^.

The mean methylation levels of CG, CHG, CHH, and total C were 87.41%, 72.75%, 8.90%, and 29.45%, respectively (Fig. 1b, Supplemental Table 1). Most CG and CHG sites were heavily methylated. In contrast, the majority of CHH sites showed relatively low methylation levels, with 80% of CHH sites presenting methylation levels of 20–60% (Supplemental Fig. 2).

### DNA methylation profiles in genes and TEs

The methylation profiles in different genomic regions were examined separately. For the genic regions, the CG sites showed the highest methylation in the gene body, decreased methylation in gene-adjacent regions, and the lowest methylation in transcriptional start/end sites (TSS/TES) (Fig. 1d). Methylation in the CHG context showed moderate levels in the gene body, increased levels in upstream regions, and slightly lower levels in downstream regions (Fig. 1d). The CHH context showed hypomethylation in gene bodies but increased methylation levels in flanking sequences (Fig. 1d). For all three sequence contexts, exons presented hypomethylation compared with introns (Supplemental Fig. 3). To further investigate whether DNA methylation affects the gene transcriptional levels, we clustered genes into four groups on the basis of their transcriptional levels. The data showed that CG methylation in gene bodies was positively correlated with gene transcriptional levels, while CG methylation around TSS/TES and downstream 2 kb regions decreased gradually with increasing gene expression levels. For the CHG and CHH contexts, the methylation levels in TSS/TES and downstream 2 kb regions were negatively correlated with transcriptional levels. However, CHH methylation in promoter regions and gene transcription levels showed a positive correlation (Supplemental Fig. 4).

Consistent with reports in most other plant species, all three cytosine contexts showed hypermethylation in TE regions but low methylation in the flanking regions (Fig. 1e). The hypermethylation of TEs is possibly responsible for the suppression of abundant TEs in the garden asparagus genome.

### Comparative analysis of DNA methylation in male and female flowers at different developmental stages

In general, female flower buds showed higher methylation levels than male flower buds in all contexts (CG, CHG, and CHH), with more remarkable differences in the CHH context (Supplemental Table 1, Fig. 1b). During asparagus flower development, the methylation levels in the meiotic stage tended to be higher than those in the premeiotic stage in both male and female flower buds. Such differences were mild at CG and CHG sites but more significant in the asymmetric CHH context (Supplemental Table 1, Fig. 1b). Accordingly, the highest methylation levels were observed in female flower buds at the meiotic stage in both genes and TEs. In contrast, male flower buds at the premeiotic stage showed the lowest methylation levels. Detailed examination revealed that the high DNA methylation levels were primarily due to increased CHH methylation (Fig. 1d, e).

**Fig. 2:**
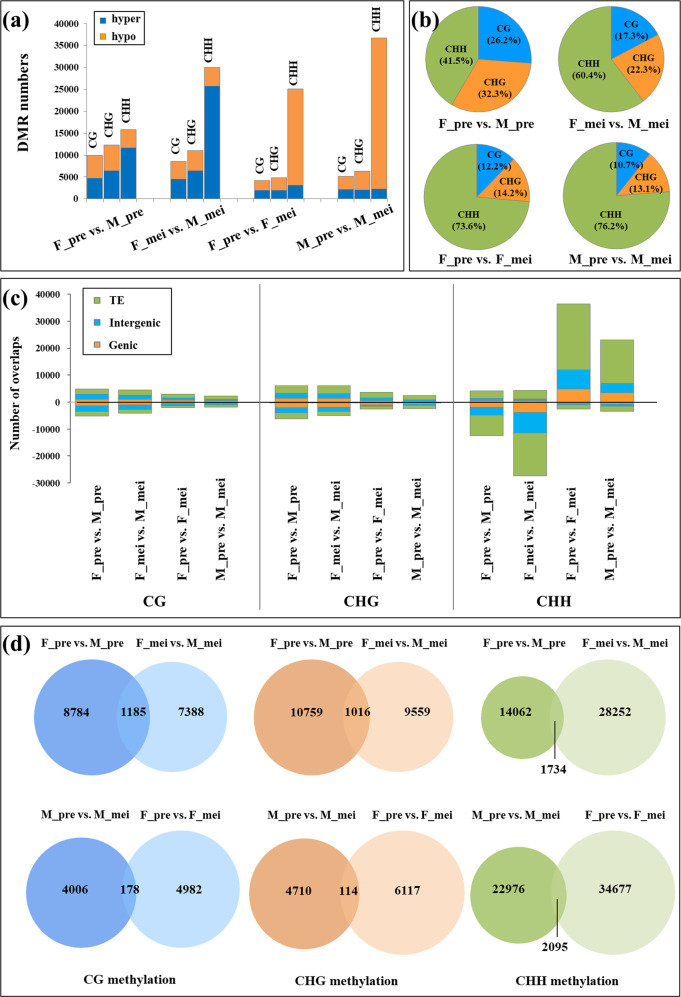
DMRs in male and female flower buds at the premeiotic and meiotic stages. **a** DMRs were identified by paired comparisons of the BS-seq patterns of the four samples (male flower buds at the premeiotic stage, M_pre; female flower buds at the meiotic stage, F_pre; male flower buds at the meiotic stage, M_mei, and female flower buds at the meiotic stage, M_mei) in each context. The DMR numbers, including the hyper- and hypo-DMRs for each cytosine context, are shown. **b** DMR proportions for all three contexts. **c** Localization of DMRs in different annotated features, including genic, TE, and intergenic regions. **d** DMRs were largely nonoverlapping in pairwise comparisons between F_pre vs. M_pre and F_mei vs. M_mei (upper). The DMRs between M_pre vs. M_mei and F_pre vs. F_mei also did not overlap (lower)

To investigate the methylation differences more specifically, we identified DMRs in a pairwise fashion (F_pre versus M_pre, F_mei versus M_mei, M_pre versus M_mei, and F_pre versus F_mei). At the premeiotic stage, we identified a total of 9969, 12,275, and 15,796 DMRs in the CG, CHG, and CHH contexts, respectively, in female flowers relative to male flowers. Among these DMRs, 15,202 were hypermethylated (hyper-DMRs), while 22,838 were hypomethylated (hypo-DMRs) (Fig. 2a). We discovered that 41.5% of the total DMRs were in CHH sites, while CHG DMRs and CG DMRs exhibited lower percentages (Fig. 2b). The meiotic stage (F_mei vs. M_mei) showed similar trends. More strikingly, CHH-DMRs were more abundant in the meiotic stage (60.4%) than in the premeiotic stage, and hypo-CHH-DMRs were the most abundant (Fig. 2a, b). For the developmental stages, pairwise comparisons of M_pre vs. M_mei and F_pre vs. F_mei yielded similar results, with much more hyper-DMRs than hypo-DMRs, and CHH-DMRs were predominant (Fig. 2a, b).

We further assessed the genomic locations of these DMRs and observed that the DMRs were predominantly located in TEs and intergenic regions for all three contexts (Fig. 2c). In addition, although the variation patterns between F_pre vs. M_pre and F_mei vs. M_mei were similar, the DMRs of all three contexts did not overlap. A similar tendency was observed between the pairwise comparisons of M_pre vs. M_mei and F_pre vs. F_mei (Fig. 2d). These results indicate that dynamic methylation patterns are potentially responsible for garden asparagus sexual differentiation and distinct flower development.

### Comparison of the transcriptomes between male and female asparagus flowers at different developmental stages

**Fig. 3:**
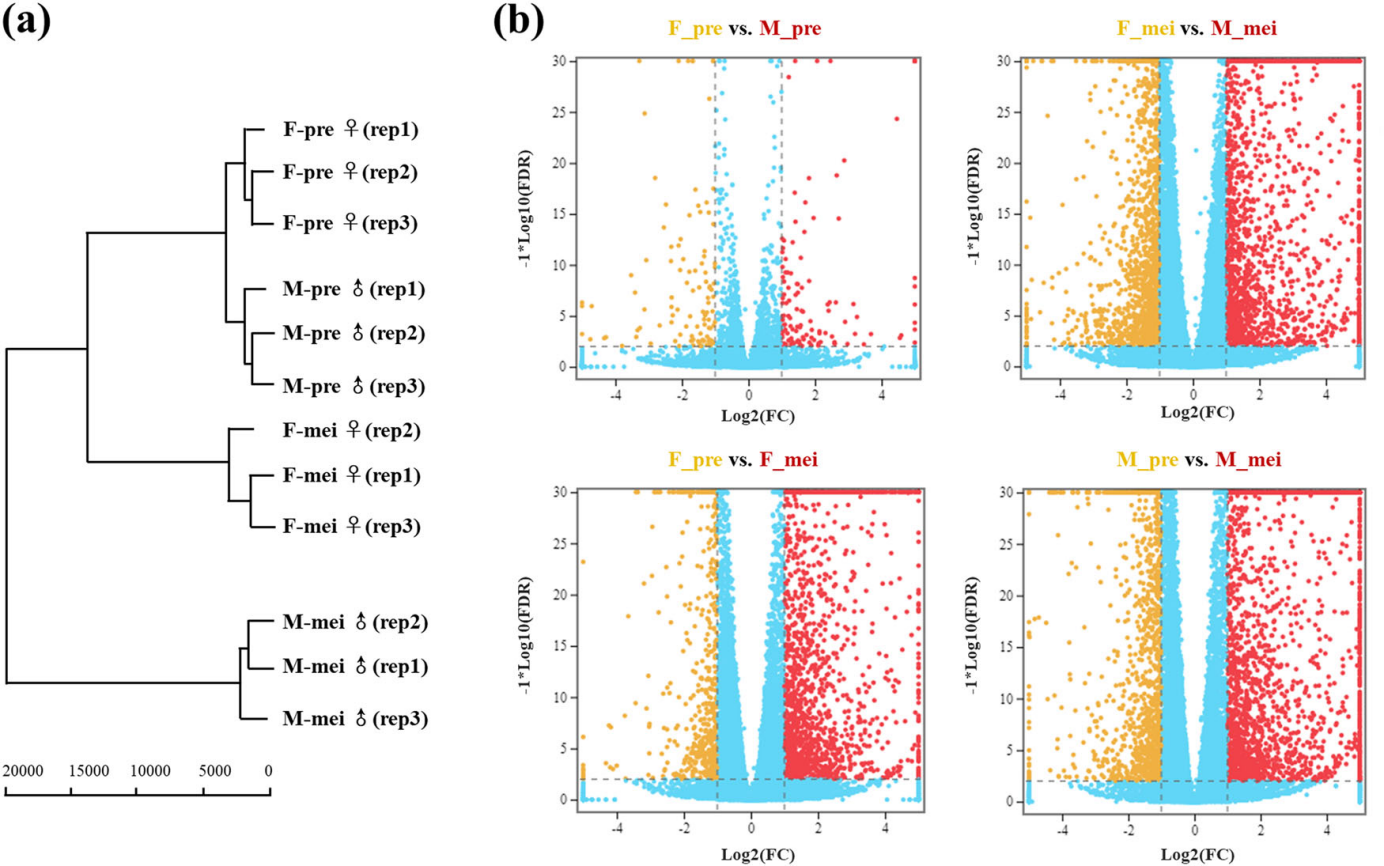
Clustering and comparison of DEGs in male and female flower buds of garden asparagus at different developmental stages. **a** Clustering of male and female flower samples at different developmental stages (based on 22,322 expressed genes). **b** Gene expression comparisons of different groups: F_pre vs. M_pre, F_mei vs. M_mei, F_pre vs. F_mei, and M_pre vs. M_mei

We generated transcriptome profiles for the same male and female flower buds to investigate the potential transcriptional consequences of methylation variations related to garden asparagus male and female flower development. Clustering analysis showed consistency among the three replicates of each sample (Fig. 3a). In particular, the flowers at the premeiotic stage from both males and females clustered together, which was consistent with their similar phenotypes (Fig. 3a). Accordingly, there were fewer sex-biased DEGs at the premeiotic stage than at the meiotic stage (Fig. 3b). The numbers of upregulated DEGs at both the premeiotic and meiotic stages were higher than the number of downregulated DEGs in males compared with females. With regard to male and female flower development, 4701 and 2708 DEGs were identified for males and females, respectively. Most of these DEGs were upregulated at the meiotic stage (Fig. 3b). Moreover, using criteria of an FPKM < 0.4 in one sex and an FPKM > 2 in the other sex, we identified 39 male-specific and 37 female-specific genes at the premeiotic stage. More sex-specific genes were detected at the meiotic stage, with 738 being male-specific and 189 being female-specific.

### DEG–DMR correlation analysis of male and female flowers at the meiotic stage

To investigate whether the sexual differentiation process in garden asparagus was associated with DNA methylation, we focused on analyzing DMR-related genes by comparing F_mei and M_mei flowers because the meiotic stage is the critical stage of sexual differentiation^24,29^. Approximately 12,999 hyper-DMRs and 36,635 hypo-DMRs were identified in M_mei flowers compared with F_mei flowers. Among them, 17,667 were distributed in the gene body or promoter/downstream regions and thus potentially contributed to gene regulation during sexual differentiation and flower development. A total of 8,199 genes were detected as sex-biased DMR-related genes in garden asparagus flower buds. Among them, 5960 were female hyper-DMR-associated genes, while 2,239 were male hyper-DMR-associated genes at the meiotic stage. Among the female hyper-DMR-associated genes, we identified 639 female-biased DEGs (cluster 1), 702 male-biased DEGs (cluster 2), and 4,619 non-DEGs (cluster 3). Among the male hyper-DMR-associated genes, a total of 208 female-biased DEGs (cluster 4), 284 male-biased DEGs (cluster 5), and 1,747 non-DEGs (cluster 6) were identified. Cluster 1 and cluster 4 genes were highly expressed in female flowers. In contrast, cluster 2 and cluster 5 genes presented higher transcriptional levels in male flowers. However, the transcriptional levels of the cluster 3 and cluster 6 genes did not show significant differences (Supplemental Fig. 5). These data suggested that the majority of DMRs did not have a significant impact on the transcriptional levels of adjacent genes.

**Fig. 4:**
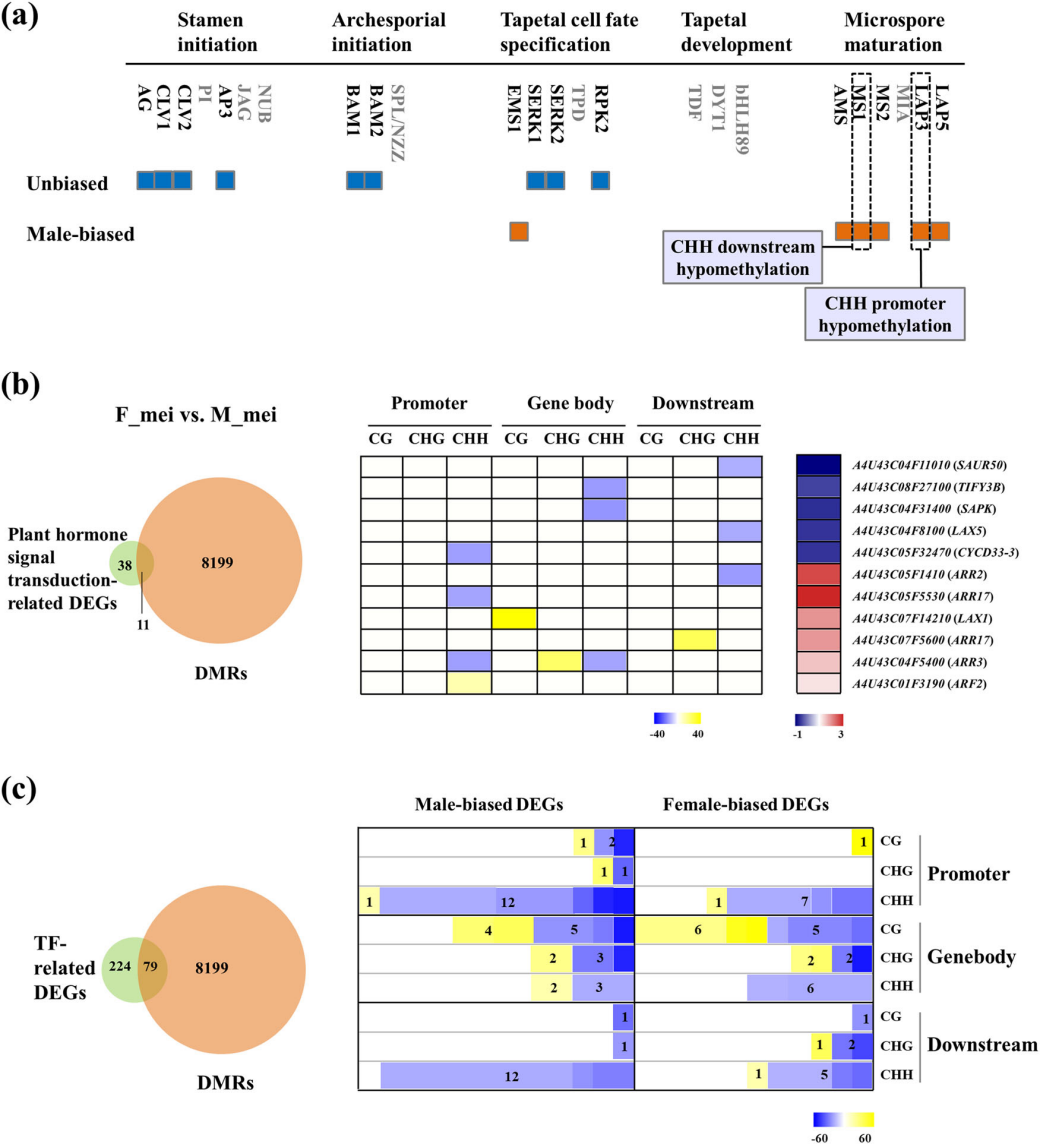
Association analysis of DEG–DMR correlated genes between male and female flowers at the meiotic stage. **a** Expression patterns and DEG–DMR association analysis of critical genes involved in the anther development pathway between male and female flowers at the meiotic stage. Genes whose names are in a gray color were unable to be successfully annotated in our transcriptome data. Genes with no sex-biased expression are presented with blue squares, whereas genes showing male-biased expression are presented with orange squares. The two male-biased genes marked with dashed square frames showed marked differential methylation between the two opposite sexes. **b** Number of DMR-associated DEGs belonging to the plant hormone signaling pathway (left) and the differential methylation profiles of male- and female-biased genes involved in the plant hormone signaling pathway (right). **c** Number of DMR-associated DEGs encoding TFs (left) and the differential methylation profiles of male- and female-biased genes annotated as TF genes (right)

We further analyzed the DMR-associated DEGs. The orthologs of the known genes involved in the anther development pathway were examined, and six genes were identified as male-biased genes, including *EMS1*, *AMS*, *MS1*, *MS2*, *LAP3*, and *LAP5* (Fig. 4a). Among these genes, two were DMR-associated DEGs. *LAP3* harbors a hypomethylated CHH region in the promoter region, and *MS1* has a CHH hypomethylation region in the downstream region. In the plant hormone signal transduction pathway, 38 genes showed sex-biased expression at the meiotic stage. Among them, 11 DEGs showed varied DNA methylation (Fig. 4b). These genes mainly belonged to auxin- and cytokinin-related signal transduction pathways. Specifically, the cytokinin-related signaling genes *AoARR2* and *AoARR3* and two *AoARR17* genes were more highly expressed in male flower buds than in female flower buds. Most of these genes presented hypomethylation at CHH sites in male flowers in comparison with female flowers (Fig. 4b).

In addition, the DMR-associated DEGs comprised numerous TF-encoded genes. Among the 224 DEGs between the two types of flowers at the meiotic stage, approximately 35.3% (79) were correlated with DMRs. Of these DMR-associated DEGs, 40 were preferentially expressed in males, and 39 were female-biased expressed genes. The differentially expressed TFs included members of the bHLH, MYB, NAC, MADS, bZIP, and other TF families. Members of the MYB and NAC families were highly enriched among male-biased DEGs, whereas members of the bHLH family were highly enriched in female-biased DEGs (Supplemental Fig. 6). Most of these genes (74.7%) showed differential methylation in the CHH context, and the CHH context DMRs were mainly hypomethylated in males. Interestingly, most CG-type DMRs (76.9%) were located in the gene body, whereas the majority of the CHH context DMRs (78%) were located in promoter or downstream gene regions (Fig. 4c). These results demonstrated that methylation dynamics at CHH sites might contribute to the sexual differentiation of garden asparagus.

### Genes potentially modulated by DNA methylation variance during unisexual flower development

**Fig. 5:**
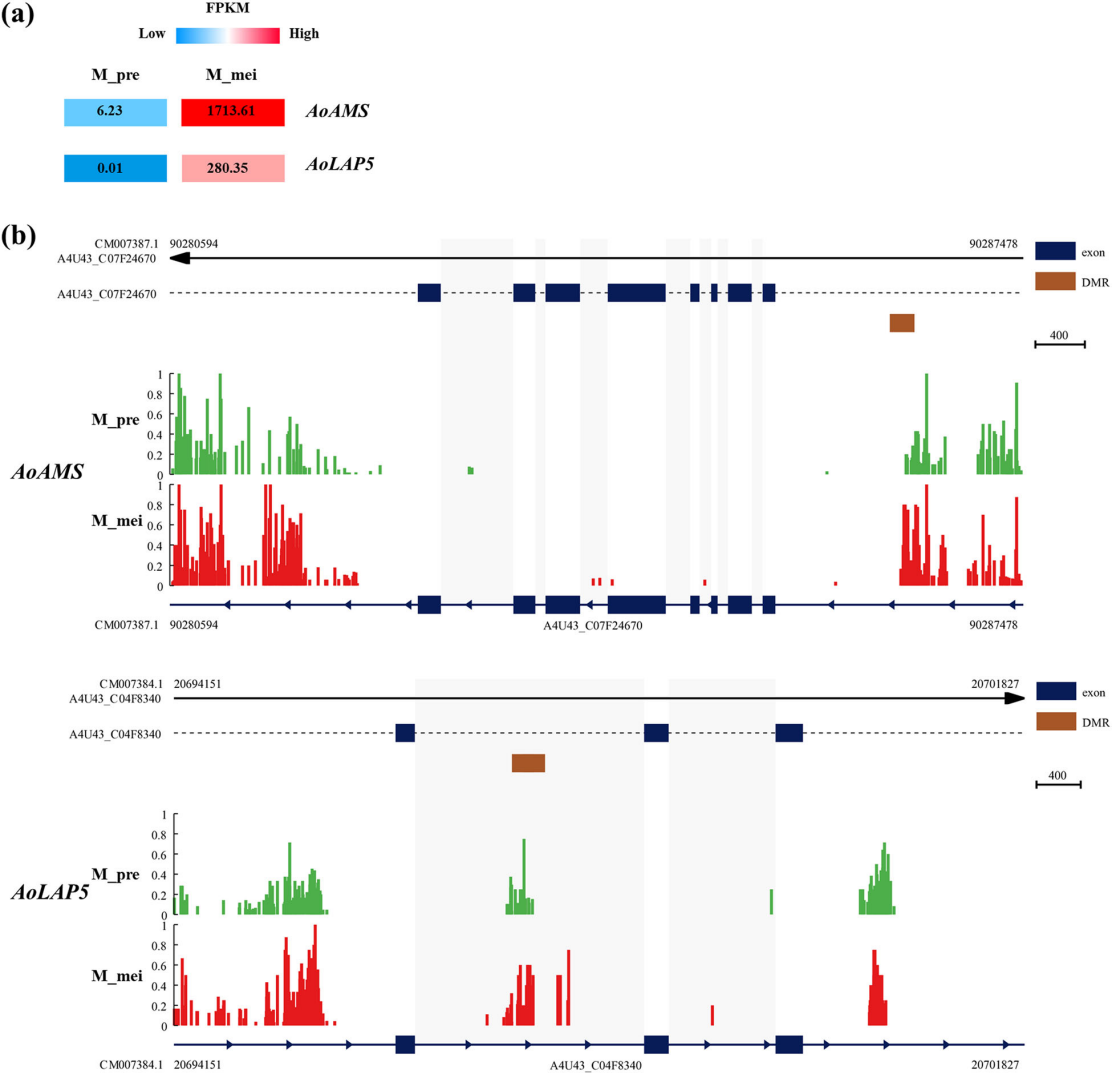
Analysis of DMR-associated DEGs involved in anther development of male flower buds between the premeiotic and meiotic stages. **a** Expression pattern of three correlated genes involved in the anther development pathway of male flower buds between the premeiotic and meiotic stages. **b** Methylation patterns of these three genes in male flower buds at the premeiotic and meiotic stages. The yellow frames indicate DMRs of M_pre vs. M_mei

To demonstrate the role of DNA methylation-related gene modulation in flower development, we conducted interrelation analysis between DEGs and DMRs for the M_pre vs. M_mei and F_pre vs. F_mei comparison groups. During male flower development, two male-biased DEGs within the anther development pathway showed varied DNA methylation. *AoAMS* harbored a hypermethylated CHH DMR in the promoter region at the meiotic stage in comparison with the premeiotic stage. The other gene, *AoLAP5*, had a hypermethylated CHH DMR in the gene body (Fig. 5). In addition, a number of DMR-associated DEGs implicated in plant hormone signal transduction and TFs were identified in the M_pre vs. M_mei (Supplemental Figs. 7 and 8) and F_pre vs. F_mei comparison groups (Supplemental Fig. 9). The majority of these genes presented hypermethylation in the CHH context in meiotic flowers compared with premeiotic flowers (Supplemental Figs. 8 and 9).

### DNA methylation did not have a significant influence on the expression variance of sex-determining genes

The Y chromosome present in males is a key determinant of sex. The MSY of garden asparagus has 13 genes. Among these genes, *SOFF* was found to suppress female function, and *TDF1* was regarded as a male promoter. Thus, the two genes were considered to be critical sex-determining genes. They specifically existed in the male genome and were absent from the female genome. *SOFF* was not expressed in male flowers at the premeiotic stage and was weakly expressed at the meiotic stage. *TDF1* was mildly expressed at the premeiotic stage in male flowers but was significantly upregulated at the meiotic stage. However, the methylation statuses of the two genes and flanking sequences did not differ between the two developmental stages (Supplemental Fig. 10). Thus, we speculated that DNA methylation might not directly influence the expression variances of sex-determining genes of garden asparagus.

### DNA methylation profiling in sex chromosomes

**Fig. 6:**
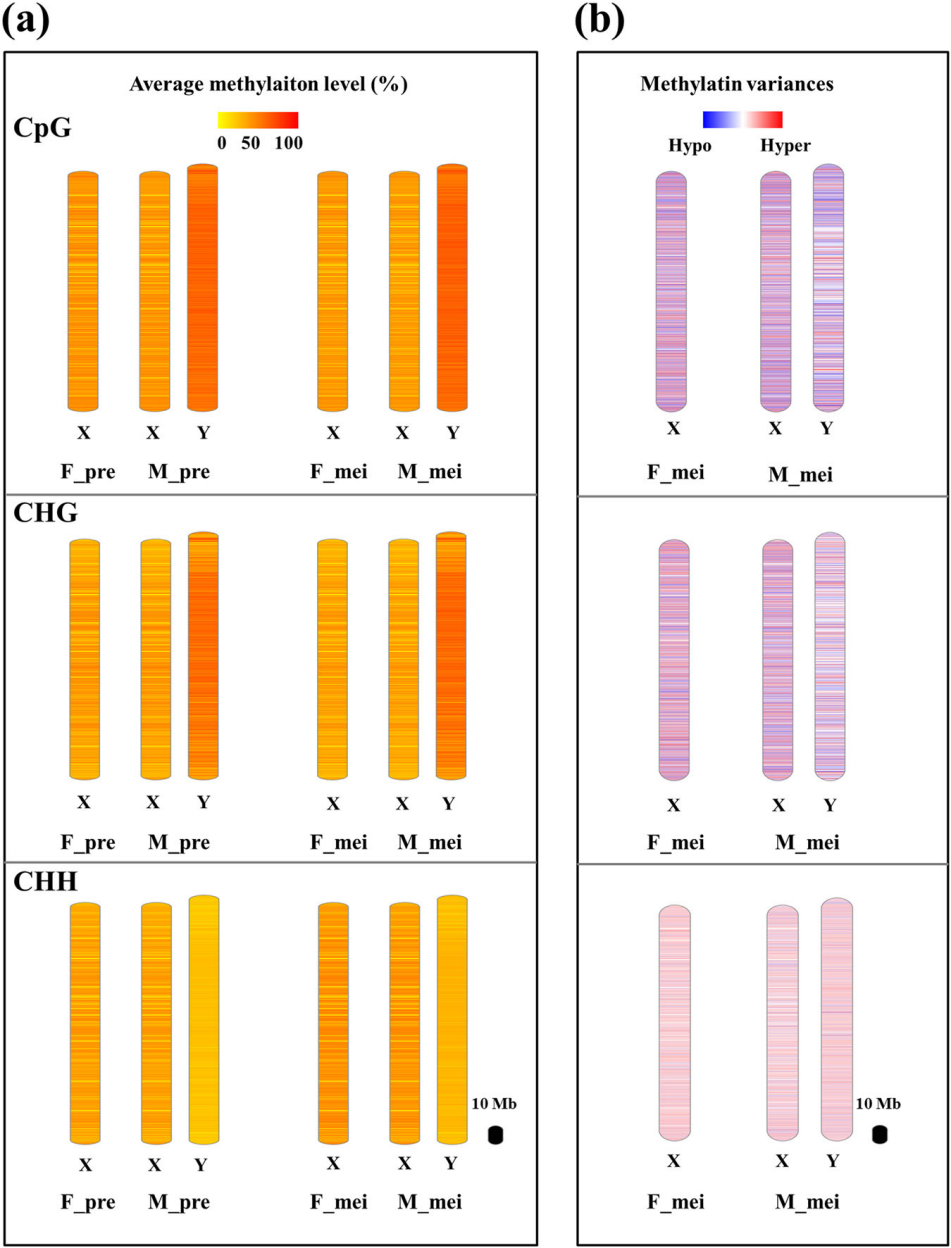
Methylation profiles of garden asparagus sex chromosomes. **a** Average methylation levels of the X and Y chromosomes in male and female flowers. **b** DMR distribution of male and female flowers at the meiotic stage

To investigate the methylation profiles of sex chromosomes of garden asparagus, we explored the methylation levels of X and Y chromosomes in flower buds (Fig. 6). We found that CpG and CHG methylation levels were apparently elevated in the majority of Y chromosomes compared with X chromosomes; that is, the Y chromosome was hypermethylated in the CpG and CHG contexts. In contrast, the CHH methylation level of the X chromosome was higher than that of the Y chromosome. In addition, the methylation distribution and levels of the X chromosome in females and males were nearly identical in all three contexts.

DMR profiling of sex chromosomes showed that Y chromosomes had wider-ranging methylation variances than X chromosomes. The DMRs in the X chromosomes showed differences between males and females, but no consistent patterns were found.

## Discussion

### Sex-biased DNA methylation in garden asparagus

Whole-genome BS-seq and subsequent comparison analyses were performed to investigate sex-biased DNA methylation in garden asparagus. Interestingly, most discovered DMRs were found in the CHH context, and the majority of DMRs were located in TE regions. The existing sex-specific DMRs reflect DNA methylation variances between male and female flowers. These sex-specific methylation variations might function in shaping sex characteristics by regulating gene transcription levels. However, we discovered that the majority of genes carrying DMRs in their gene bodies or flanking regions did not present significant changes in their expression levels. In fact, this phenomenon has also been discovered in other studies on the relationships of DNA methylation with a number of biological processes^37–39^. For example, one study investigated the influence of epigenetic modification on soybean domestication and evolution by comparing and analyzing the variation in DNA methylation in several soybean cultivars and related materials. A total of 5,412 DMRs were identified; however, co-differential expression analyses suggested that merely 22.54% of DMRs were associated with varied gene expression levels^39^. Interpretation of these results is complicated because although tremendous advancements in DNA methylation have been achieved in recent years, much DNA methylation remains enigmatic.

### DNA methylation may not be involved in shaping the expression profiles of sex-determining genes in garden asparagus

Previous studies have revealed that DNA methylation may participate in sex determination in several dioecious plants. Treatment with the hypomethylating chemical 5-azacytidine in *Silene* induces a sex alteration in some male individuals, suggesting that the sex-determining process involves DNA methylation in *Silene*^40^. In hexaploid persimmon (*Diospyros kaki*), male buds have a higher methylated promoter of *MeGI* than female buds; *MeGI* is autosomal gene-regulating another fertility and can be targeted by the sex-determining gene *OGI*. DNA methylation inhibition induces the conversion of immature male buds into female flowers^41^. Aside from these findings, epigenetic modulation of sex determination has not been discovered in other dioecious plants. Dioecy has independently evolved many times in plants. Thus, the sex determination mechanisms are distinct in different plants, even in closely related species. For example, the epigenetic sex regulation mechanism in hexaploid persimmon is remarkably divergent from the genetic determination of its close relative diploid persimmon^41^. In garden asparagus, during male flower development, the expression levels of the two sex-determining genes were increased, whereas the DNA methylation levels of the two genes and adjacent regions were not significantly changed. The results indicate that DNA methylation may not have strong influences on the transcriptional variances of sex-determining genes of garden asparagus. It is well known that sex is stable in garden asparagus and little affected by external factors. This is in agreement with the fact that sex determination of garden asparagus may not involve epigenetic regulation. However, our knowledge of the molecular mechanisms of these sex-determining genes in garden asparagus is rather limited. We look forward to further functional studies elucidating the modulation mechanisms of these sex-determining genes and confirming the relationship between DNA methylation and sex determination.

### DNA methylation and sex chromosome evolution of garden asparagus

Sex chromosomes evolved from pairs of autosomes through a gradual and continuous process including a number of key events, such as sex-determining gene formation, recombination suppression, repetitive sequence accumulation, and Y chromosome degeneration^14,42^. DNA methylation has been considered a crucial modulator involved in the whole evolutionary process of sex chromosomes^43^. However, few studies have been conducted to support this view^44^. Garden asparagus harbors “young” homomorphic sex chromosomes, which are suitable for the investigation of the events and mechanisms of early sex chromosome evolution^45^. During the sex chromosome evolution process, repetitive sequence accumulation and heterochromatization are believed to have facilitated suppression of recombination and sex chromosome differentiation^46^. In fact, DNA methylation represents a defense mechanism to suppress TEs and other types of invasive DNA^47,48^. DNA methylation facilitates the shaping of heterochromatin^49,50^, and it is regarded to contribute to recombination suppression of sex chromosomes in papaya^51^. As has been reported previously in *Silene latifolia*, which has large heteromorphic Y chromosomes, TE accumulation may lead to hypermethylation of the Y chromosome^15^. In this study, we found that the methylation levels in the CpG and CHG contexts of Y chromosomes were apparently higher than those of X chromosomes. These results indicated that DNA methylation might function in suppressing recombination between the sex-specific regions of the X and Y chromosomes in garden asparagus.

### Possible epigenetic regulation of genes involved in the anther development pathway and their possible roles in sexual differentiation and male flower development in garden asparagus

Flowering, the most essential developmental process in higher plant life, consists of a complicated network involving a diverse range of gene interactions^52,53^. In dioecious plants, sex-determining genes may modulate downstream genetic networks and other pathways to mediate unisexual flower development^54^. We compared the methylation levels and gene expression in male and female flower buds of garden asparagus at the premeiotic and meiotic stages. At the premeiotic stage, the male and female flower buds showed no morphological differences. Comparative transcriptome analysis also presented a small number of DEGs. However, the sex-determining gene *TDF1* was expressed, albeit at a low level. The results suggested that sexual differentiation was initiated at this stage. At the meiotic stage, a large number of DEGs were identified. The sex-determining gene *TDF1* showed much higher expression at the meiotic stage than at the premeiotic stage, and *SOFF* also showed a higher expression level, implying that the meiotic stage is a critical sexual differentiation stage. Among the DMR-associated DEGs between females and males at the meiotic stage, *MS1* and *LAP3* are critical genes involved in the anther development pathway of *Arabidopsis thaliana*. In addition, association analysis between male flowers at different stages showed that *AMS* and *LAP5* were involved in the anther development pathway. Thus, epigenetic regulation of *MS1*, *LAP3*, *AMS*, and *LAP5* might mediate garden asparagus flower development and sexual differentiation. *MS1* encodes a protein similar to the PHD-finger motif TF and plays a significant role in male gametogenesis and another development. Homozygous *ms1* mutants of *A. thaliana* fail to produce functional pollen^55^. *AMS* encodes a bHLH-type TF that is essential for male fertility. It can also bind to the promoter regions of a set of anther development genes and regulate their expression^56^. The other two genes (*LAP3* and *LAP5*) encode calcium-dependent phosphotriesterase superfamily proteins and chalcone/stilbene synthase family proteins, respectively. They are both involved in pollen exine formation^57,58^. Thus far, no studies have reported the epigenetic regulation of these four genes. However, many genes participating in the anther development pathway are mediated by DNA methylation^59,60^. Interestingly, we found that these genes with higher expression in male flowers at the meiotic stage than in female flowers at the meiotic stage or male flowers at the premeiotic stage all harbored hypomethylation in the CHH context. These results indicate that the functions of *AoMS1*, *AoLAP3*, *AoAMS*, and *AoLAP5* in garden asparagus sexual differentiation and flower development potentially involve epigenetic regulation.

### Plant hormone signaling genes and TF genes under epigenetic regulation are likely associated with sexual differentiation and flower development in garden asparagus

Plant hormones are essential regulators that exert profound and diverse effects on almost the entire plant development process^61^. Reports have shown that plant hormone signaling genes are involved in the modulation of sex determination and sexual differentiation of several dioecious plants^22,62^. In *Actinidia*, the cytokinin response modulator *SyGI*, which acts as a dominant suppressor of female development, has been identified as a potential sex determinant gene^22^. Importantly, DNA methylation has been found to contribute to the plant hormone signaling process of sexual expression in papaya^38^. In this study, we found that male and female flowers exhibited obvious methylation variance in the number of DEGs involved in plant hormone signal transduction. Most of these DEGs belonged to cytokinin- and auxin-response pathways. In particular, *AoARR2*, *AoARR3*, and two *AoARR17* genes showed higher expression in male flower buds than in female flower buds. The *ARR* gene family is related to cytokinin signaling^63^. It is known that cytokinin signaling can interact with typical MADS-box proteins to influence early inflorescence development^64^. Studies in *Arabidopsis* have shown that *AtARR16* coupled with *AtAHP6* may restrict cytokinin signaling and participate in gynoecium development^65^. *PbRR9*, a homologous gene of *AtARR16* and *AtARR17* at the sex-determining region, has been proposed to have an epigenetically mediated role in sex determination in *Populus balsamifera*^62^. The high expression levels of *AoARR* genes in male flowers might contribute to inflorescence differentiation and androecium formation in garden asparagus. This is in contrast with the situation in papaya, in which a *CpARR5* gene associated with hypomethylation at its downstream region is downregulated in male flowers. These results demonstrate that the mechanisms of sexual differentiation differ in different dioecious plants. However, these results confirm that plant hormone-mediated transcription regulation associated with epigenetic modulation indeed plays a role in the sexual differentiation and flower development of these dioecious plants.

TFs are the main regulators of sexual differentiation and flower development processes in plants. Indeed, some genes within the anther development pathway and the plant hormone signaling process discussed above encode TFs. For example, AMS is a member of the bHLH TF family. In addition, a number of co-differentially expressed TF genes with DMRs were identified by comparing male and female flowers in this study. Among the various TF families, MYB and NAC were enriched in male flowers, whereas bHLH was enriched in female flowers. Numerous studies have shown that epigenetic regulation of TF genes plays important role in sex differentiation in animals ^66,67^. For example, in zebrafish, elevated temperature induces masculinization by reducing the expression levels of two TF genes, *sox9b,* and *esr1*, via DNA methylation^67^. Epigenetic regulation of TF genes has also been confirmed to participate in sex determination/differentiation and flower development in some plant species^68,69^. It has been reported that epigenetic changes in the promoter of *CmWIP1*, a C2H2 zinc-finger TF gene in melon, can repress the transcription of *CmACS7*, inhibit gynoecium development and lead to staminate flowers^70^. In *Arabidopsis*, the MADS-box gene *FLF* (for *FLOWERING LOCUS F*) can repress flowering regulated by vernalization and DNA methylation^71^. We speculate that the identified co-differentially expressed TF genes with DMRs might be associated with garden asparagus sexual differentiation and unisexual flower development. The characterization and future analysis of key genes that participate in plant hormone signaling and genes encoding TFs will be helpful in understanding the complicated network of sexual differentiation in garden asparagus.

## Supplementary information

Supplemental material

## Data Availability

All the raw sequencing data reported in this paper have been deposited in the Genome Sequence Archive under accession number CRA004058 and are publicly accessible at https://bigd.big.ac.cn/gsa.
