# Supplementary material for: DNA methylation is involved in sexual differentiation and sex chromosome evolution in the dioecious plant garden asparagus
Source: Hortic Res. 2021 Sep 1;8:198. doi: 10.1038/s41438-021-00633-9 (PMC8408194; doi:10.1038/s41438-021-00633-9)
Supplement: Supplementary file 1 — Supplemental material [file 41438_2021_633_MOESM1_ESM.docx]

**Table S1 Summary of BS-seq results and estimation of methylation levels**

| **Sample** | **Read no.** | **Uniquely mapped** | **Mapped ratio, %** | **%mCG** | **%mCHG** | **%mCHH** | **Total %mC** | **Coversion rate, %** | **Sequencing depth** |
| --- | --- | --- | --- | --- | --- | --- | --- | --- | --- |
| M_pre | 263159174 | 230080470 | 87.43 | 87.41 | 72.75 | 8.90 | 29.45 | 99.89 | 29.06 |
| M_mei | 262371408 | 228790506 | 87.20 | 88.10 | 74.29 | 11.26 | 31.93 | 99.93 | 28.90 |
| F_pre | 264221480 | 230787210 | 87.35 | 87.55 | 72.98 | 9.53 | 30.11 | 99.91 | 29.15 |
| F_mei | 264698940 | 230698045 | 87.15 | 88.96 | 75.70 | 12.23 | 33.22 | 99.93 | 29.14 |

M_pre represents male flower buds at pre-meiotic stage; M_mei represents male flower buds at meiotic stage;

F_pre represents female buds at pre-meiotic stage; F_mei represents female flower buds at meiotic stage.


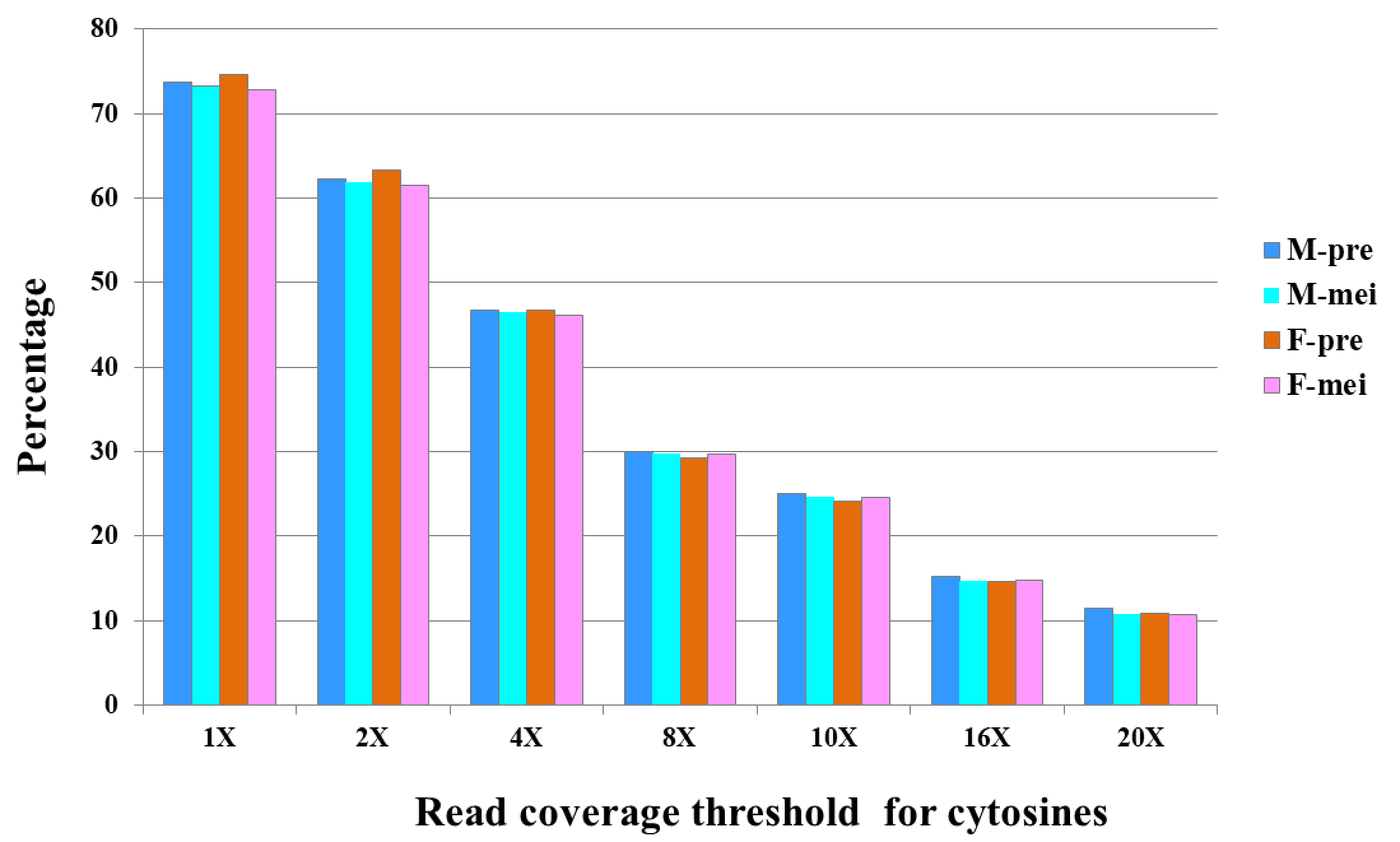


**Supplemental Fig. 1 Coverage depth of BS-seq.**


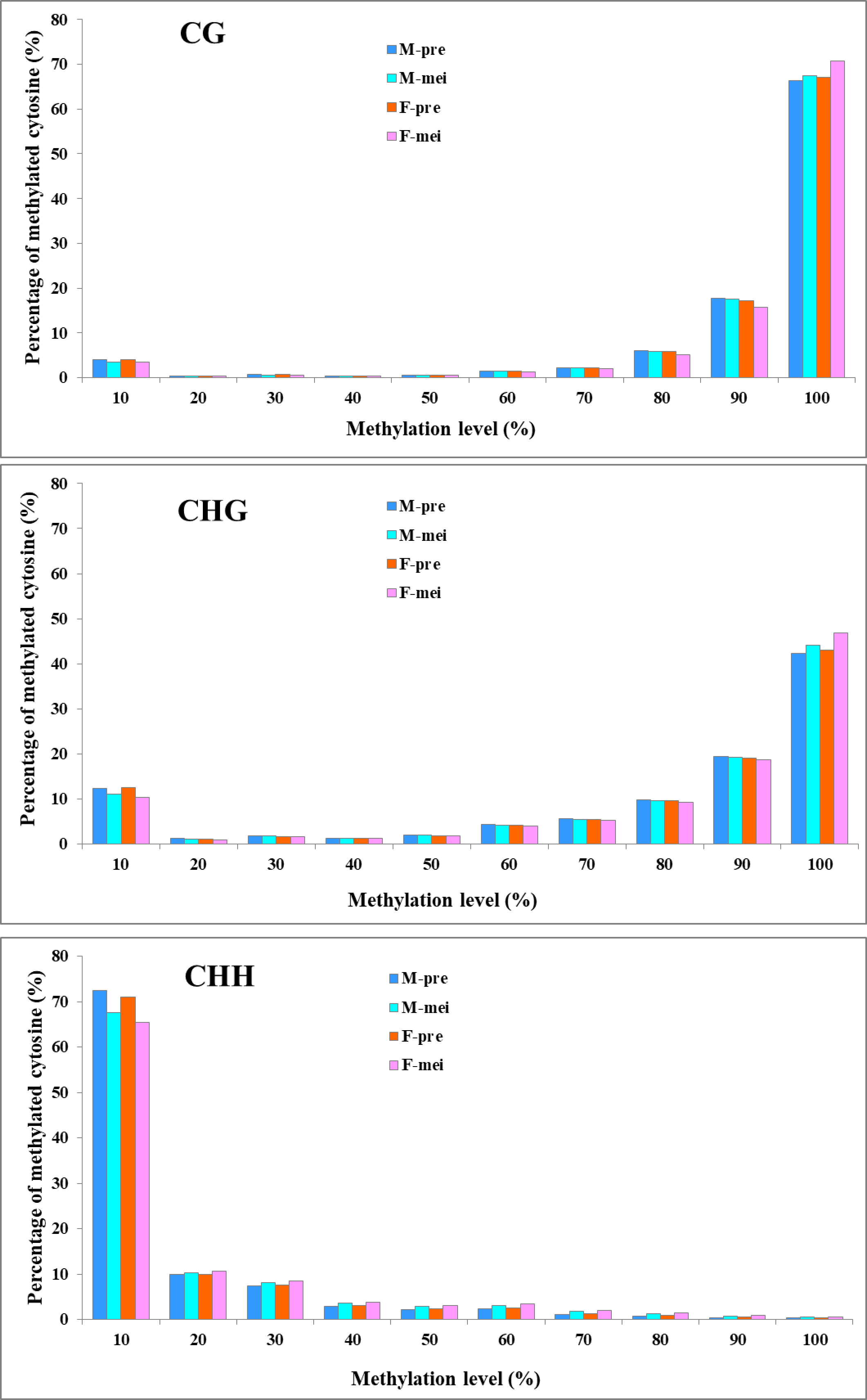


**Supplemental Fig. 2 Distribution of the methylation levels in different sequence contexts for male and female flowers at the pre-meiotic and meiotic stages.**


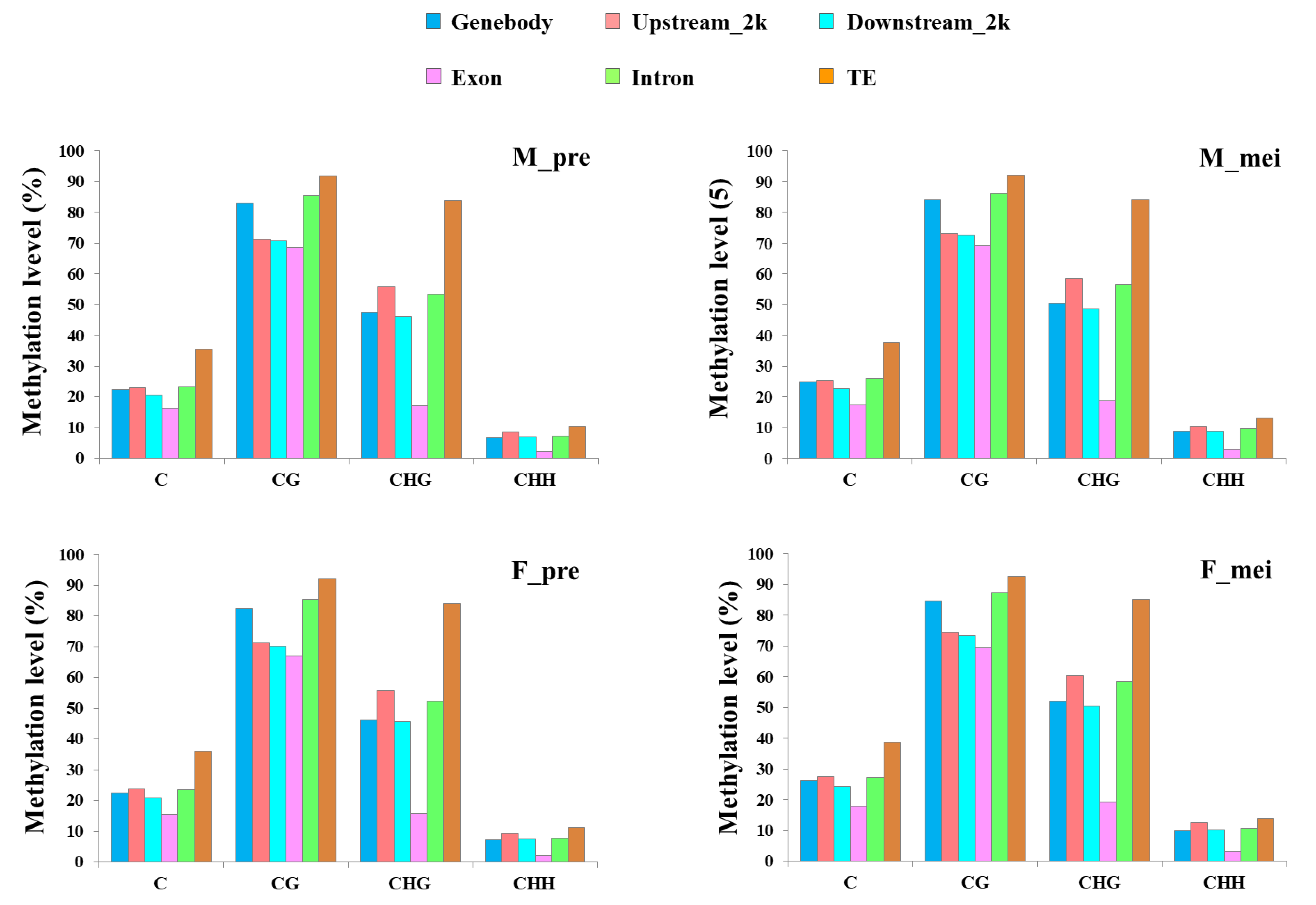


**Supplemental Fig. 3 DNA methylation levels among different genic features.**


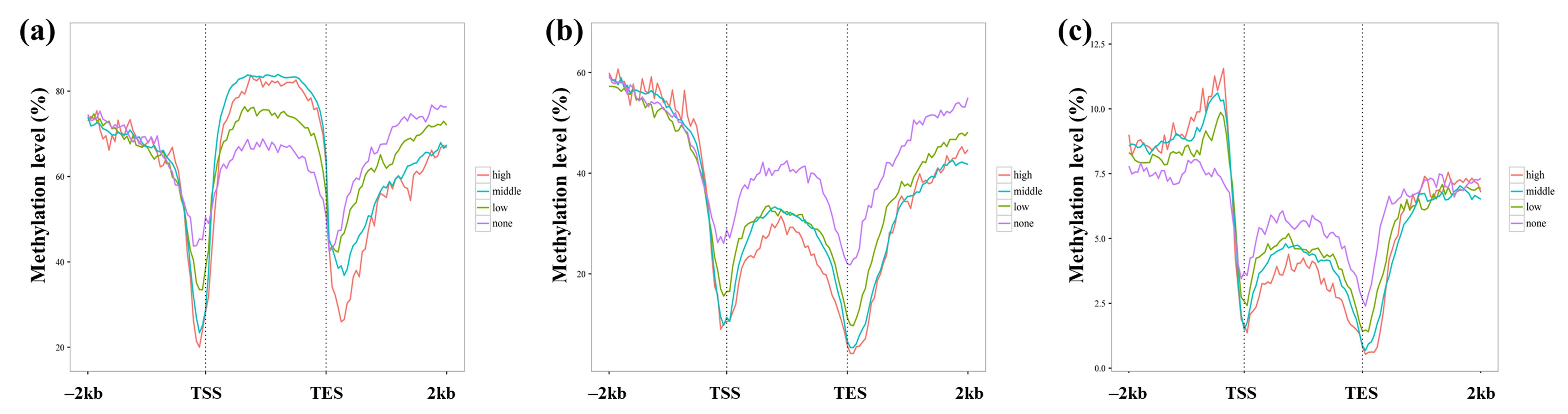


**Supplemental Fig. 4 Correlation analysis between DNA methylation and gene expression in CG, CHG, and CHH sequence contexts.**


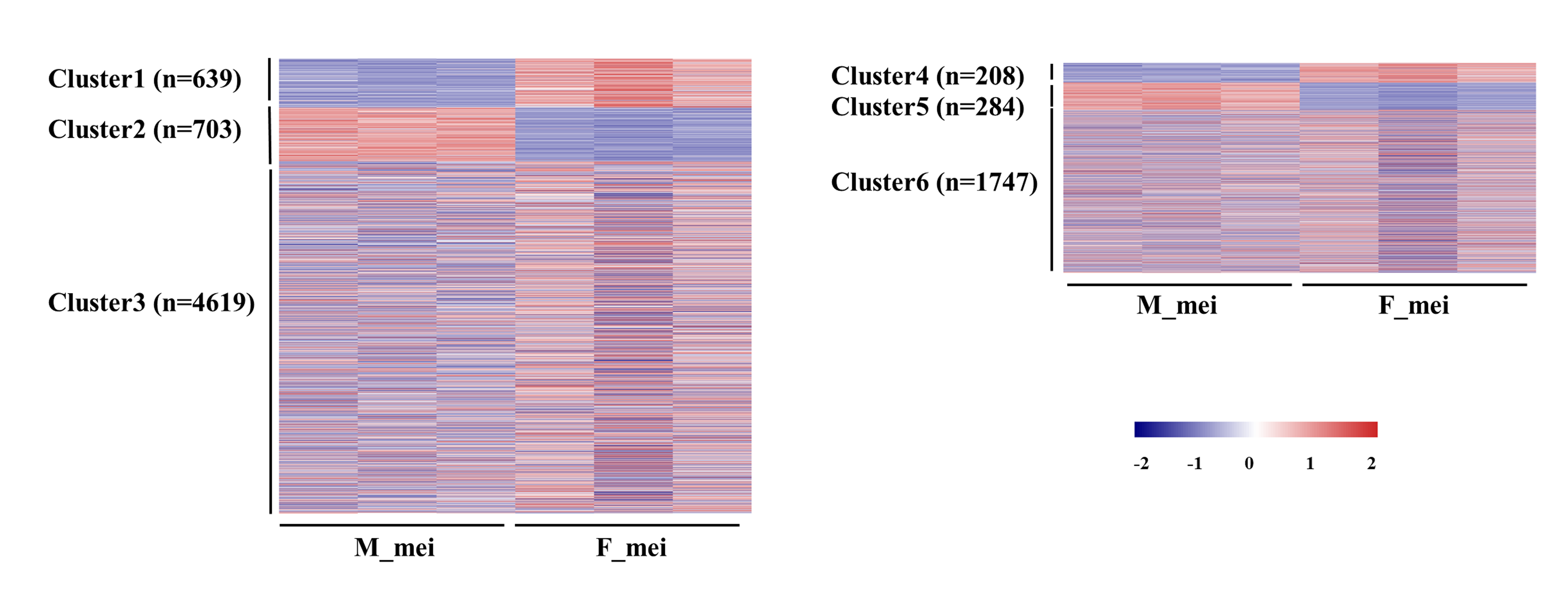


**Supplemental Fig. 5 Heatmaps presenting the expression variations of the six clusters of DMR-related genes between male and female meiotic flowers.**


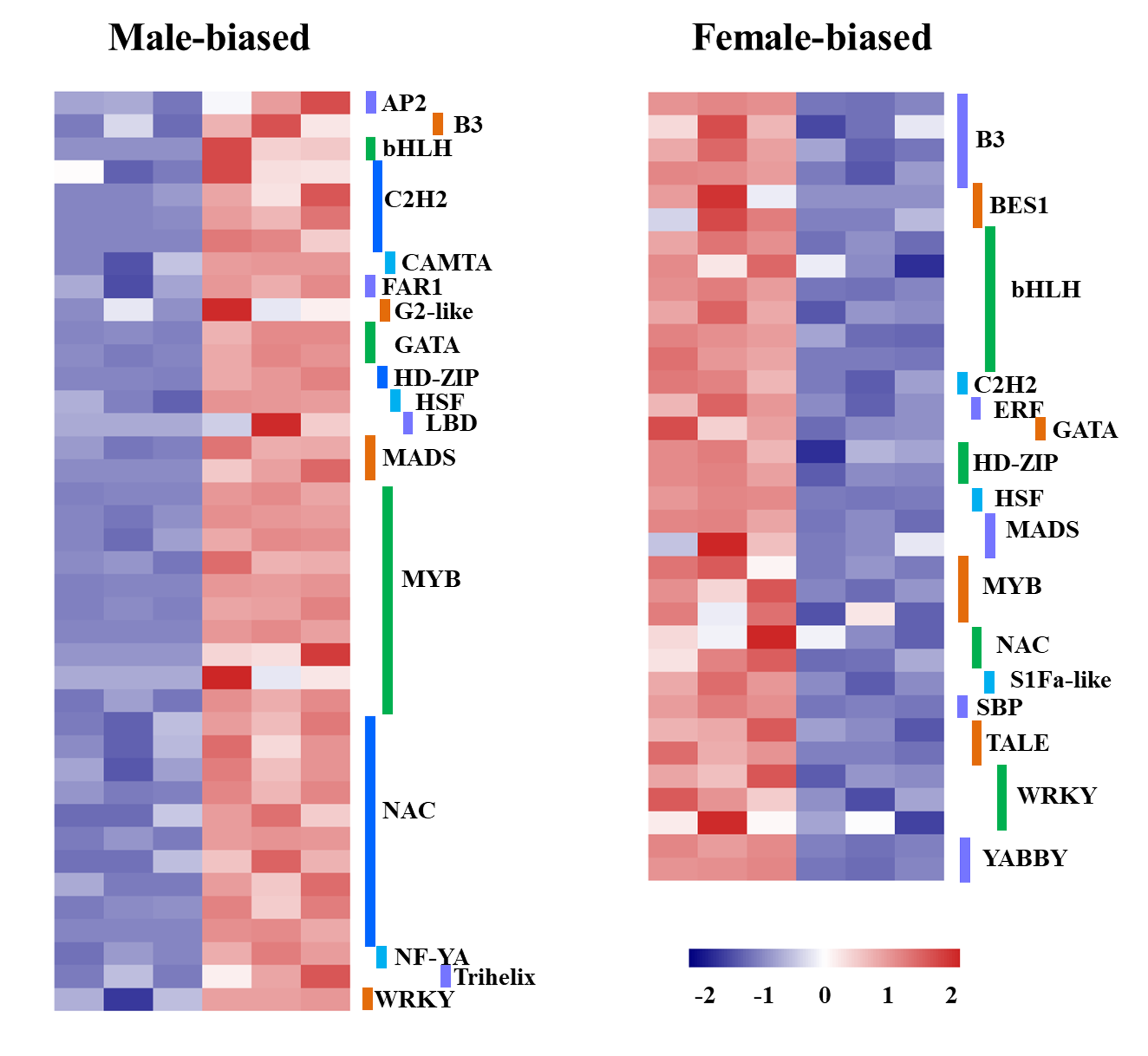


**Supplemental Fig. 6 Heatmap diagram showing differential expression of TF genes that showed differential methylation level between male and female meiotic flowers.**


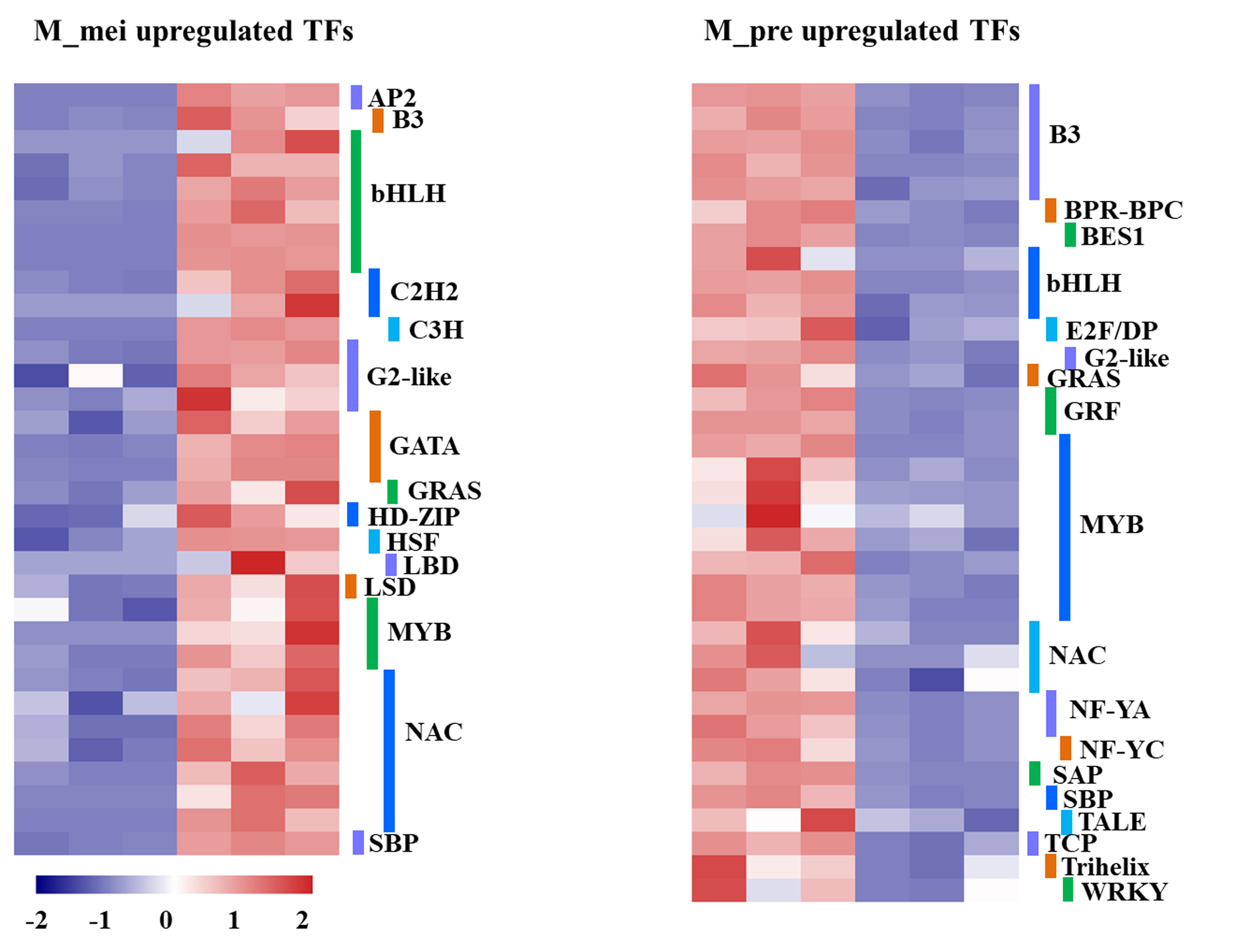


**Supplemental Fig. 7 Heatmap diagram showing differential expression of TF genes that showed differential methylation level between pre-meiotic and meiotic male flowers.**


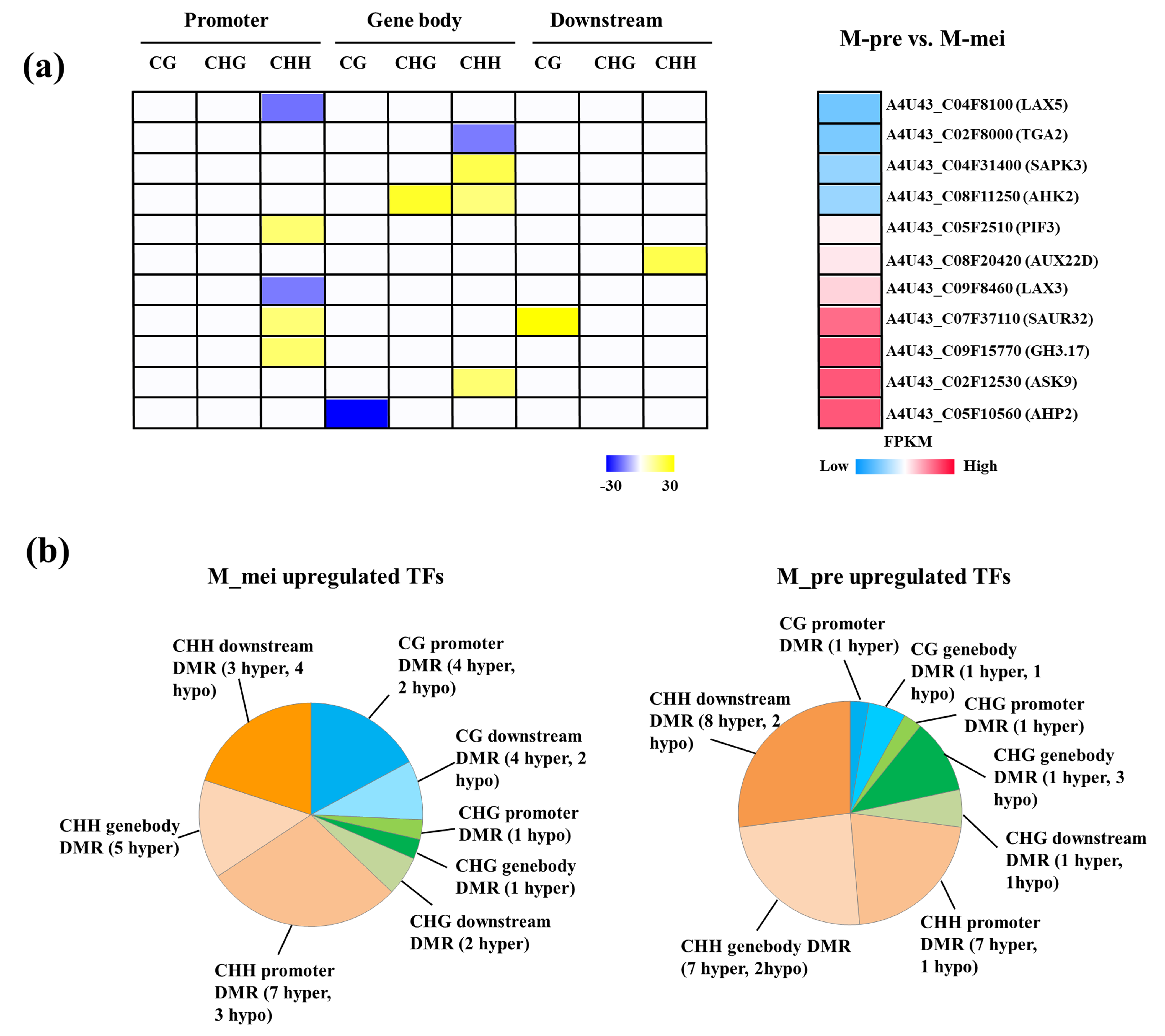


**Supplemental Fig. 8 Association analysis of DEG–DMR correlated genes of male flower buds between pre-meiotic and meiotic stages.** (a) Expression and differential methylation profiles of DEG–DMR correlated genes involved in the plant hormone signaling pathway during male flower development. (b) Differential methylation profiles of DEG–DMR correlated genes encoding TFs during male flower development.


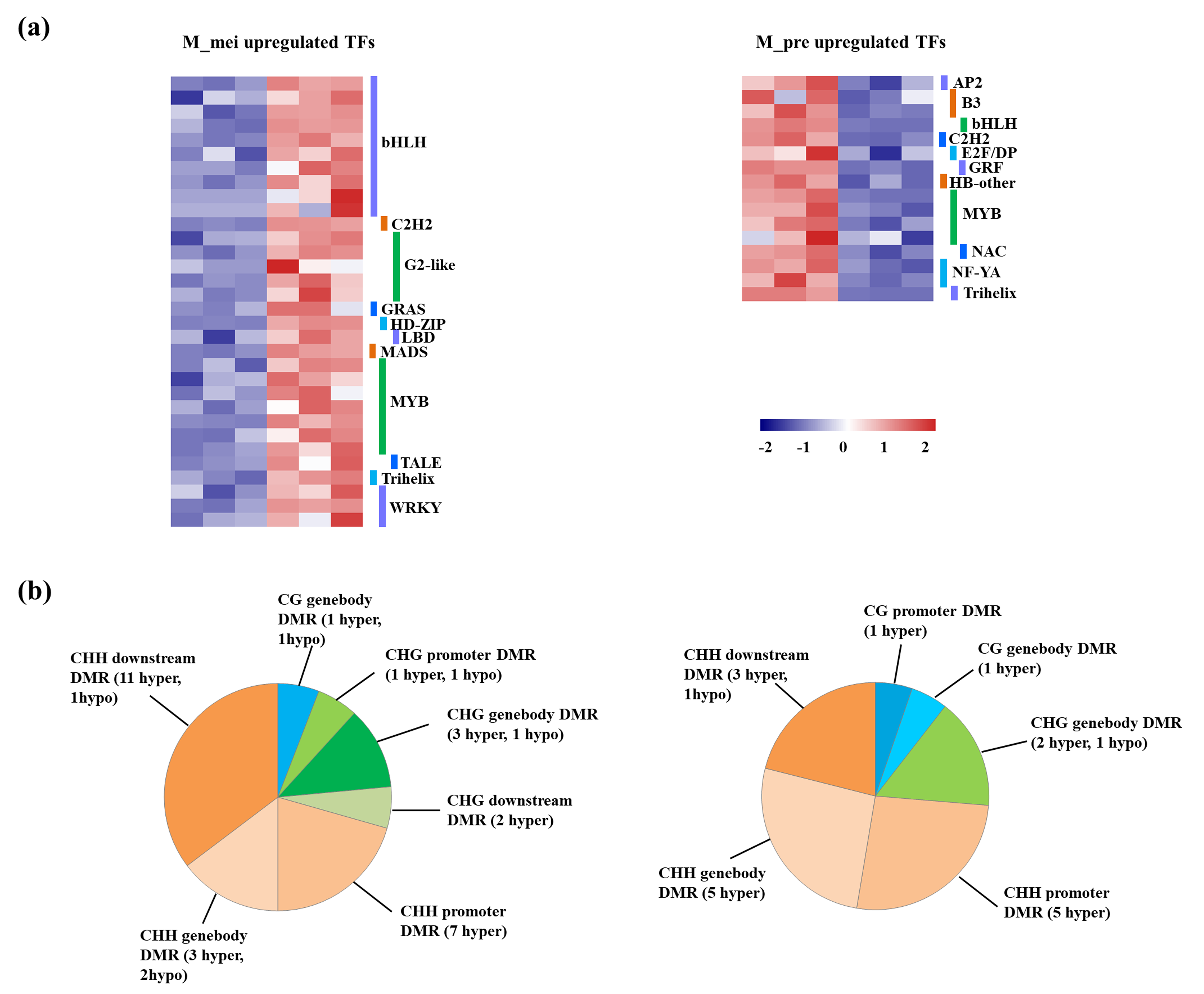


**Supplemental Fig. 9 Association analysis of DEG–DMR correlated genes of female flower buds between pre-meiotic and meiotic stages.** (a) Expression and differential methylation profiles of DEG–DMR correlated genes involved in plant hormone signaling pathway during female flower development. (c) Differential methylation profiles of DEG–DMR correlated genes encoding TFs during female flower development.


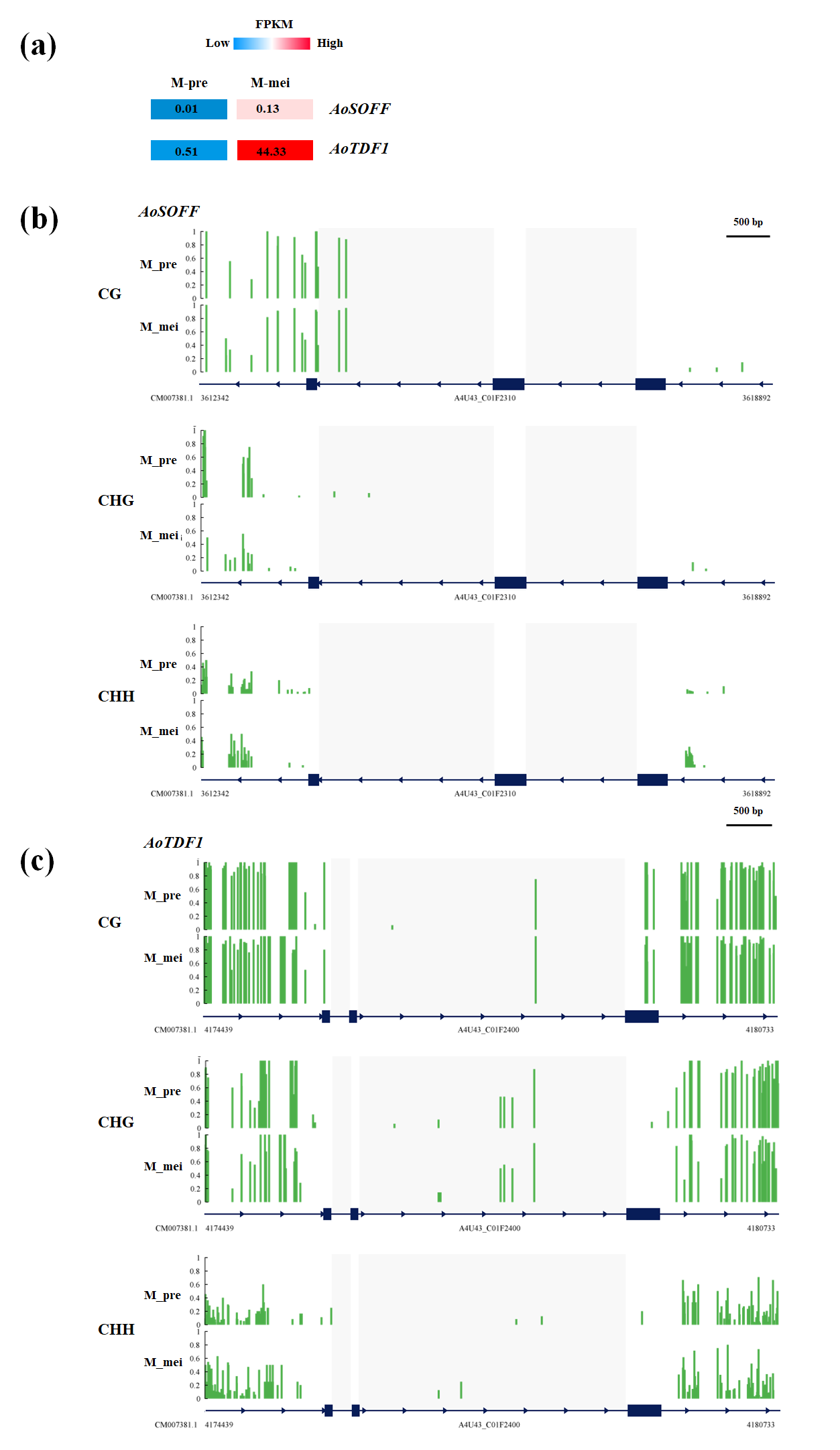


**Supplemental Fig. 10 Expression and methylation variations of sex-determining genes *AoSOFF* and *AoTDF1*.** (a) Expression patterns of *AoSOFF* and *AoTDF1* of male flower buds between pre-meiotic and meiotic stages. (b) Methylation patterns of the two genes in male flower buds at the pre-meiotic and meiotic stages.
